# 3D Bioprinting in Skin Related Research: Recent Achievements and Application Perspectives

**DOI:** 10.1021/acssynbio.1c00547

**Published:** 2021-12-30

**Authors:** Anna Olejnik, Julia Anna Semba, Adam Kulpa, Aleksandra Dańczak-Pazdrowska, Jakub Dalibor Rybka, Justyna Gornowicz-Porowska

**Affiliations:** † Faculty of Chemistry, Adam Mickiewicz University in Poznań, Uniwersytetu Poznańskiego 8, 61-614 Poznań, Poland; ‡ Center for Advanced Technology, Adam Mickiewicz University, Uniwersytetu Poznańskiego 10, 61-614 Poznań, Poland; § Faculty of Biology, Adam Mickiewicz University in Poznań, Uniwersytetu Poznańskiego 6, 61-614 Poznań, Poland; ∥ Department of Dermatology, Poznan University of Medical Sciences, Przybyszewskiego 49, 60-356 Poznań, Poland; ⊥ Department and Division of Practical Cosmetology and Skin Diseases Prophylaxis, Poznan University of Medicinal Sciences, Mazowiecka 33, 60-623 Poznań, Poland

**Keywords:** bioequivalents, three-dimensional skin bioprinting, bioinks, skin substituents, bioprinting methods, 3D bioprinters

## Abstract

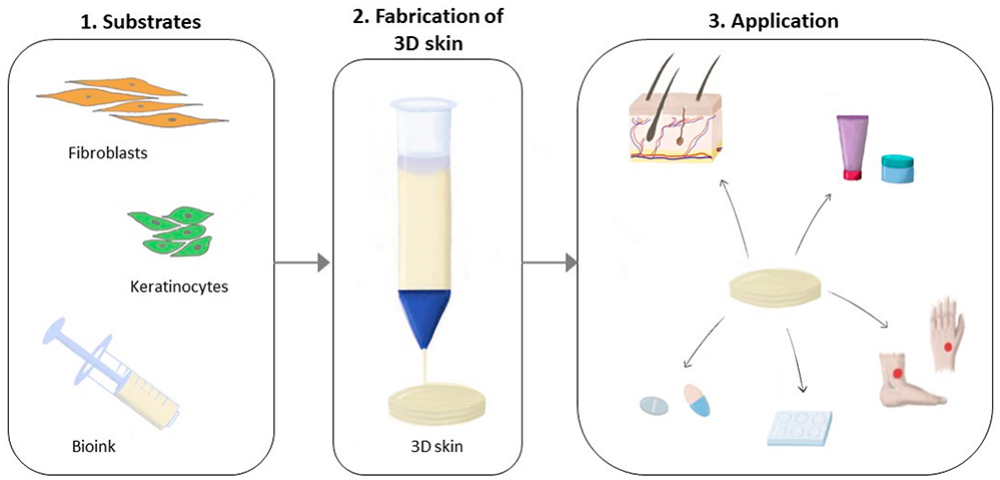

In recent years, significant progress has been observed in the field of skin bioprinting, which has a huge potential to revolutionize the way of treatment in injury and surgery. Furthermore, it may be considered as an appropriate platform to perform the assessment and screening of cosmetic and pharmaceutical formulations. Therefore, the objective of this paper was to review the latest advances in 3D bioprinting dedicated to skin applications. In order to explain the boundaries of this technology, the architecture and functions of the native skin were briefly described. The principles of bioprinting methods were outlined along with a detailed description of key elements that are required to fabricate the skin equivalents. Next, the overview of recent progress in 3D bioprinting studies was presented. The article also highlighted the potential applications of bioengineered skin substituents in various fields including regenerative medicine, modeling of diseases, and cosmetics/drugs testing. The advantages, limitations, and future directions of this technology were also discussed.

## 1 Introduction

Over the past decade, 3D bioprinting has gained worldwide significant attention from scientists involved in biological, medical, and pharmaceutical studies. In the beginning, it is essential to understand the difference between 3D printing and 3D bioprinting. In the first technique, layers of materials (plastics, metal, polymer resins, rubber) are created to obtain a three-dimensional structure. It is used to manufacture 3D-shaped objects. This technology has found applications in various fields including medicine, dentistry, engineering, architecture, agriculture, aerospace, and product design.^1−3^ In the medical area, it serves to produce anatomical models, implants, prosthetics, therapeutic devices, surgical instruments, specialized tools, and 3D plastic models that assist surgeons in operations.^4,5^ In radiology, patient-specific physical three-dimensional models can be designed from medical images that enable us to solve and analyze surgical problems.^6^ The possibility to use data from computed tomography or magnetic resonance imaging is the appreciable advantage in preoperative planning of complex operations, in particular in transplantology, oral and maxillofacial surgery, or congenital heart disease.^7−9^ The clinical trials in preoperative planning were also registered in orthopedics and maxillofacial surgery.^10^ Likewise, there is activity to print synthetic, personalized implants and patient specific instruments. Moreover, 3D printing is useful to recognize visible abnormalities and confront them with imaging techniques.^4^ In turn, bioprinting is an innovative technology that is applied to obtain three-dimensional complex structures using cells, biomaterials, and biological molecules.^11,12^ In simple terms, bioprinting functions in a similar way to standard 3D printing; however, the conventional ink is replaced by bioink that comprises cells and biomaterials required to form tissue constructs with a high degree of repeatability, flexibility, and accuracy.^11,13^ Due to the computer-driven bioprinters, the cells and biomaterials can be deposited precisely in order to achieve the predefined structures. Generally, three stages can be distinguished in bioprinting. Initially, precise information about tissues/organs should be collected to select appropriate materials and to define models. Second, the information is transferred into an electrical signal to provide the control under the printer to fabricate the tissues. In the last step, the stable structure is developed.^14−17^ 3D bioprinting belongs to the Additive Manufacturing technology that may have a broad spectrum of applications including tissue engineering,^18^ transplantation,^16^ drug screening, cancer research,^19^ cardiovascular and regenerative medicine,^20^ as well as dentistry.^21^ This method can be applied to regenerate the tooth-like composite tissues and enables us to control their shapes. Furthermore, bioprinting was also used to regenerate cartilage and bones.^22,23^

This technology also gives the opportunity to fabricate skin by using selected types of cells. Up until now, a skin equivalent that contains all skin elements has not been printed. However, the technology is still in the developing stage. The bioprinted skin constructs were first fabricated by Lee and collaborators in 2009, who added human dermal fibroblasts to a collagen hydrogel.^24^ At the same time Koch et al.^25^ focused attention on bioprinting skin equivalents by adding to collagen bioink keratinocytes and fibroblasts. In 2010, Binder et al. applied for the first time the 3D inkjet-printer skin substitutes using human fibroblasts and keratinocytes to repair wounds.^26^ Since that time, significant progress in this field has been observed. The aim of this paper is to review the latest advances in 3D bioprinting dedicated to skin applications.

## 2 Skin Architecture and Function

**Figure 1:**
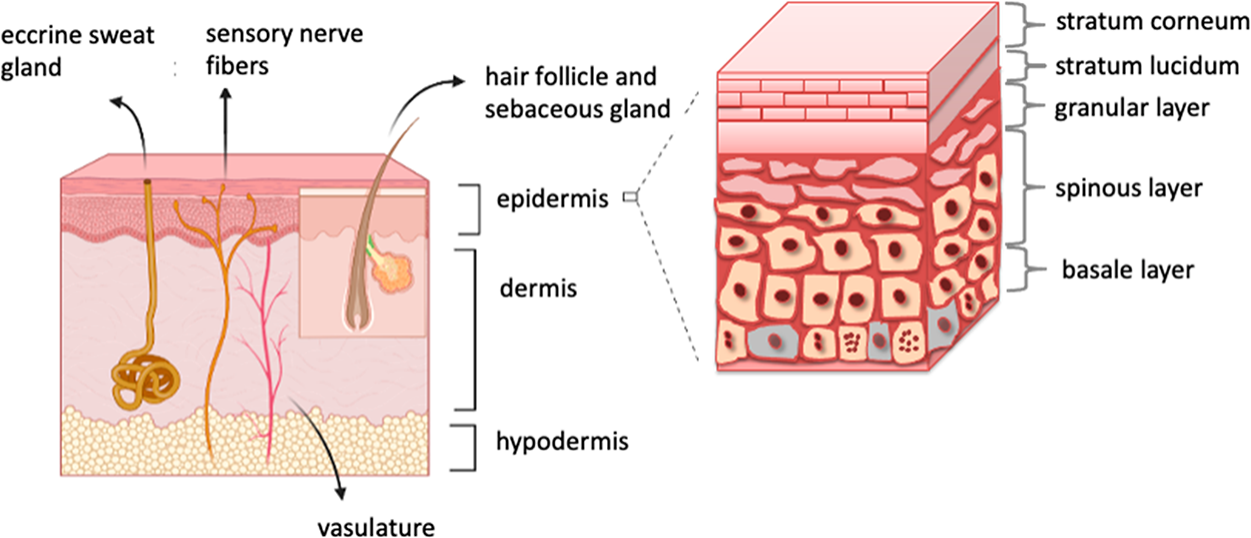
Schematic structure of the skin: the stratum corneum (the outmost layer), the viable epidermis, and the dermis.

The skin is the largest organ of the human body, which is characterized by multidimensional architecture. It consists of unique, structurally different layers with specific properties: epidermis, dermis, and hypodermis (Figure 1). The skin is responsible for many vital functions which are compartment-dependent; however, skin layers often act synergistically.^27−33^ Thus, one of the key problems of skin fabrication using bioprinting techniques is not only to deposit the skin layers but also to precisely reproduce a biomimetic tissue.^34^ The epidermis is the outermost layer of the skin. It is a stratified structure composed of several well-defined layers: basal (which is a germinal layer), spinous, granular, and stratum corneum. The latter is the result of the maturation and differentiation of keratinocytes, which account for 95% of all epidermal cells. The enucleated, densely packed keratinocytes of the stratum corneum, called corneocytes, are surrounded by a lipid matrix and form a “brick and mortar” structure, which is the main component of a proper epidermal barrier protecting against external insults (biological, physical, chemical) and restricting water loss. However, it should be stressed that keratinocytes are also a part of immunological defense. Other epidermal cells which play an important role in skin physiology include melanocytes (pigment-producing cells responsible for the protection of mitotically active cells from UV damage) and Langerhans cells (antigen-presenting cells that have a key role in the adaptive immune response).

There is a dermo-epidermal junction between the epidermis and the dermis made of proteins and proteoglycans. It is involved in the signaling between cells and in cell migration during the healing process. The dermis is a fibrous connective tissue made up of fibers (mainly collagen and elastic in smaller amounts), various cells (of which fibroblasts are the most numerous, but also some others like mast cells, histiocytes, or dendrocytes can be found), and a ground substance (with high water binding capacity). It is worth emphasizing that contrary to the epidermis, the dermis is largely acellular. Besides its role in adaptive and innate immunological defense, the dermis is responsible for the mechanical strength, resilience, and elasticity of the skin. Additionally, unlike the epidermis, the dermis houses blood and lymphatic vessels, several kinds of nerve endings, and appendages (apocrine and eccrine sweat glands as well as the complex structures called pilosebaceous units). Nerve endings are responsible for one of the most important functions of the skin, which is receiving stimuli from the environment. The eccrine glands together with blood vessels play a role in thermoregulation. Sebum from sebaceous glands creates a lipid film at the epidermal surface, thus enhancing the function of the epidermal barrier. The innermost layer of the skin is the subcutaneous tissue, which consists mainly of adipocytes and connective tissue septa. Their role includes insulation, mechanical cushioning, and energy storage, but they are also immunologically active.^35,36^

In the end, inhabiting microbiota together with the correct skin structure play an integral role for optimal barrier function, pathogen defense, and tissue repair with the production of essential anti-inflammatory and antimicrobial molecules to maintain skin homeostasis.^37^ Eventually, future perspectives of skin biofabrication should include research on ecosystems of obtained equivalents. The skin disruptions and declining microbial diversity may be linked to allergic as well inflammatory skin diseases. As described above, the complete architecture and function of the skin depend on all layers and their microstructure, which determine the skin’s proper function. In light of this, obtaining a tissue-engineered skin equivalent reflecting biomechanical properties seems to be a real challenge.

## 3 Bioprinting

Bioprinting is a promising technique for the commercial manufacturing of tissue constructs for regenerative medicine. This method utilizes a computer-controlled three-dimensional (3D) printer for the precise depositing of bioinks composed of viable cells, biomaterials, and additional biological substances in a layer-by-layer manner.^38^ The bioprinted cell-laden scaffolds aimed to promote and support new tissue formation by providing a suitable environment for cell migration, proliferation, differentiation, and ensuring proper ECM secretion. Furthermore, this technique enables the creation of constructs that mimic the architecture of patient-specific spatial geometry with the control position of cells similar to native tissue structure.^39^ There are even attempts to create a methodology for in situ skin bioprinting.^40,41^

### 3.1 3D Bioprinting Methods

There are three main techniques of 3D bioprinting, which were compared in Table 1. The most popular one is extrusion bioprinting that applied pneumatic pressure or mechanical pistons for continuous deposition of bioinks.^42,43^ In skin tissue engineering, it is also the most widely used method. It is characterized by high printing speed, affordability, and scalability of printed models. Extrusion bioprinting allows using wider types of biomaterials since high viscous materials can be utilized. However, the clogging of the nozzle is a frequently observed problem.

**Table 1:** Comparison of Methods Applied in Skin Bioprinting^47−50^

method	printing process	accuracy	pros	cons	ref.
Extrusion bioprinting	line by line	medium-low	low cost, simplicity, printability of high cell density and highly viscous bioinks	clogging nozzles, mechanical stresses generated while bioink deposition	(34,51−53)
Inkjet-based biopritning	drop by drop	medium	low cost, high cell viability, high resolution, high throughput, noncontact printing	limited bioink, low strength, nozzle clogging, risk of exposing cells to mechanical and thermal stress, possibility of cell agglomeration and sedimentation	(28,41,54,55)
Laser-assisted bioprinting	drop by drop	high	high cell viability, noncontact, nozzle-free, high precision and resolution	low scalability, low flow rate caused by fast gelation, time-consuming	(28,56,57)

Another technology applied in skin construct production is inkjet-based bioprinting.^44,45^ The technique uses a drop-on-demand printing mode usually by utilization of thermal or piezoelectric effects. In thermal bioprinting, a small heater in the printhead uses high temperatures to generate vapor bubbles within the bioink.^46^ These bubbles create the pressure pulse that extrudes bioink. In the second approach, the piezoelectric actuator converts the applied voltage into the deformation of a crystal.

These changes produce the pressure required for the drop ejection. The bioink for inkjet bioprinting should have low viscosities that affect the mechanical properties of final scaffolds.^11^ Nevertheless, this method is fast and relatively cheap. Lastly, laser-assisted bioprinting (LAB) is also applied for skin biofabrication.^56,58,59^ This is a noncontact, nozzle-free method where a laser beam is absorbed by the ribbon that generates a local bubble in bioink on the opposite side. LAB is applied for bioprinting with high cell density bioinks at a resolution of nearly a single cell. The final constructs can be printed in three different forms such as cell-suspensions, cell-encapsulated hydrogels, or cell-free models.^60^

### 3.2 Bioink

The bioink formulation is a pivotal step as its composition and structure affect the phenotype of the developing tissue.^11,39^ The mechanical and physical properties of bioink need to ensure printability and correspond to engineering tissue. The biodegradation rate of bioink should be adjusted to the cell capacity to remodel the extracellular matrix (ECM), while the products of degradation cannot be toxic or immunogenic. Despite the growing number of biomaterials used in bioprinting, only a subset of them is suitable for skin bioprinting. These biomaterials are briefly described below.

#### 3.2.1 Collagen

Collagen is the most abundant protein in the mammalian ECM and, hence, it is widely used in tissue engineering.^39^ It has excellent biocompatibility with low immunogenicity and toxicity. There are 28 types of collagen present in vertebrates.^61^ Collagen type I makes up most of the protein mass in the connective tissues of mammals; hence, it is frequently utilized for bioink production. Unfortunately, the main limitations of collagen use are its low mechanical stability, poor solubility, cost, and fibrotic tissue formation. Neutralized collagen solution heated to a temperature of 20–37 °C self-assembles into a physically cross-linked hydrogel that provides structural and biological support for cells.^62,63^ However, collagen gelation at physiological temperatures is slow so it is frequently mixed with other biomaterials. Collagen type I-based bioink has been used for extrusion skin bioprinting.^24,45^ In these studies, the collagen layers and the cell layers (fibroblast and keratinocytes) were printed separately. The printed model retained form, shape, and was morphologically and biologically similar to human skin tissue. In addition, constructs were cultured at the air–liquid interface to promote epidermal maturation.^45^

#### 3.2.2 Gelatin

Gelatin, an irreversibly denatured form of collagen, is frequently used for bioink formulation instead of collagen. Gelatin retains many similar features of collagen including cell adhesion sites and cytocompatibility; however, it has a significantly lower price and better water solubility than collagen.^64^ Gelatin is unable to form long fibrils.^65^ Instead, local regions of triple helices on different gelatin strands interact to form physical cross-links that are responsible for gelation at lower temperatures (below 30 °C).^65^ Hence, the viscosity of gelatin-based bioinks can be easily changed by altering the temperature and concentration of gelatin. The application of gelatin-based bioinks for skin tissue engineering showed promising results in the promotion of epithelialization and granulation in the wound healing process.^67^ However, the gelation of gelatin is a thermoreversible process, so its bonds are easily broken in a physiologic environment. Hence, gelatin is frequently blended with alginate for bioink production.

#### 3.2.3 Alginate

Alginate, the most popular biomaterial used for 3D bioprinting, is a linear and negatively charged polymer composed of two uronic acid monomers.^68^ This material has low toxicity and is cheap and nonimmunogenic. Alginate lacks cell and protein binding properties, so the addition of extra positively charged biomaterials is required to achieve cell adhesion.^69,70^ Alginate-based bioinks are cross-linked by divalent cations, which is described by the “egg-box” model.^71^ The most popular cross-linking solution is CaCl_2_.^39,72^ This cross-linking method is fast and heterogeneous, but is hard to bioprint. Hence, as mentioned previously, alginate is mixed with other materials, like gelatin. In terms of skin fabrication, the alginate/gelatin bioink with proper rheological parameters was also proposed.^70^ This bioink composition is subjected to two-step polymerization, namely thermal and ionic.

#### 3.2.4 Chitosan

Chitosan is a deacetylated derivative of natural chitin present in the exoskeleton of invertebrates and fungi.^73^ Chitosan is a biodegradable, biocompatible, and hemostatic polymer, which can be modified as an antimicrobial and anti-inflammatory agent for wound healing patches.^73,74^ Various physical and chemical methods can be applied for chitosan cross-linking. Chitosan has been widely used for skin tissue engineering where it has shown a positive influence on the proliferation and adhesion of keratinocytes and fibroblasts in constructed models.^75^ Nevertheless, it suffers from weak mechanical properties and slow gelation time. Therefore, it is preferred that it should be combined with the other polymers or cross-linked.^76^ The chitosan-based bioink cytocompatibility and toxicity toward human fibroblasts and keratinocytes were tested in terms of in vitro and in vivo skin tissue regeneration in rats.^77^ The results proved chitosan biocompatibility. Moreover, chitosan showed a beneficial influence on the regeneration of wounds in a rat model.

#### 3.2.5 Fibrin

Fibrinogen is a protein found in blood and has shown unique characteristics as a hemostatic agent and structural support for wound healing.^78^ It has also shown excellent biocompatibility and has a natural cell-binding site. Fibrinogen can be enzymatically converted by thrombin to fibrin. In recent years, fibrin has been used as an additive for bioinks for skin bioprinting. The diluted plasma-derived fibrin showed higher expression of type I and III collagen in keratinocytes and fibroblasts and improved cell adhesion in a printed model of skin.^64^ In the case of skin bioprinting, as an example, the fibrinogen/collagen bioink with fibroblasts and keratinocytes was engrafted in wounds on mice and pigs.^41^ This construct showed a dermal composition and accelerated re-epithelialization. Interestingly, vascular formation in regenerated tissue was observed.

## 4 Types of Cells Applied in Skin Bioprinting

Commercially available cell lines for fibroblasts, keratinocytes, melanocytes, and hair follicles are commonly applied in skin bioprinting.^34^ Furthermore, it is also possible to isolate the specific cell phenotypes from skin biopsies. Cell cultures are usually used to generate the millions of cells required for bioprinting.

So far fibroblasts have been widely applied to develop 3D-bioprinted skin constructs.^79−82^ These cells are essential for dermal formation and wound healing. In the presence of proper stimuli such as transforming growth factor beta β-1, platelet-derived growth factor, and insulin-like growth factor (IGF-1), they synthesize ECM. The majority of publications report 3D skin equivalents comprise usually two types of cells such as keratinocytes (human epidermal keratinocytes),^45^ or keratinocytes and fibroblasts. Human dermal fibroblasts were the most frequently involved in the bioprinting process.^41,45,83−86^ However, T3T mouse fibroblasts^87−89^ and L929 mouse fibroblasts^90^ were also used in some studies.

In order to mimic the natural skin, it is important to incorporate melanocytes that produce melanin, a pigment that provides photoprotection. Min et al.^91^ introduced these cells into the full-thickness skin model. Initially, a dermal layer composed of collagen and fibroblasts was printed. Afterward, the melanocytes and keratinocytes were successively bioprinted on the top of the dermis. The histological analysis confirmed the presence of melanocytes in the epidermal layer recognized as freckle-like pigmentation. Recently, more attempts have been performed to introduce melanocytes into skin models by 3D bioprinting.^92−94^

Up to now, the progress in bioprinting of blood and lymphatic vessels has been limited. These systems can be found in the dermis and are crucial for the appropriate transfer of oxygen and nutrients. In spite of their significance, there are only several articles that presented the combination of fibroblasts with endothelial cells and pericytes.^95−98^ Baltazar et al.^95^ produced multilayered vascularized skin using two types of bioinks to form the dermis and epidermis. The first one contained human foreskin dermal fibroblasts, endothelial cells, and placental pericytes. The second one constituted human foreskin keratinocytes. Other research groups applied human fibroblasts, keratinocytes, pericytes, and induced pluripotent stem cell-derived endothelial cells to fabricate skin equivalents.^97^ Li et al.^70^ employed in their studies Wharton’s jelly mesenchymal stem cells and amniotic epithelial cells, while Nocera et al.^89^ involved epithelial Vero cells in their research. Kim et al.^96^ fabricated a perfusable vascularized 3D skin model made up of the epidermis, dermis, and hypodermis. In should be mentioned that the cells that can cause skin disease can also be introduced to the biomaterials. This kind of tissue containing pathogenic cells can be applied to perform research on pathophysiology skin disorders.^45^ It should be stressed that in order to obtain the appropriate environment for cell/tissue growth the knowledge regarding cell membrane composition should be taken into account while designing 3D bioprinted skin models. It has been presented by Ferreri and Chatgilialoglu that dermatological problems strictly correlate with the functions of cell membranes.^99,100^ Well-balanced composition of fatty acids in cell membranes is crucial for their proper fluidity, permeability, hydration, and skin aging.^99^ The importance of this aspect, when cultured cells are applied, was also demonstrated by Symons et al.^101^

## 5 The Required Properties of Bioprinted Skin

The bioprinted skin should fulfill the special functional and compositional features. It should be biocompatible and should have required mechanical properties and appropriate surface chemistry. The ideal skin model should be able to transfer nutrients and reduce wound exudates.^11^ In order to reproduce the native skin, the bioprinted equivalent of the appropriate cells (keratinocytes, melanocytes, Merkel and Langerhans cells, fibroblasts, adipocytes) should be accurately deposited at certain locations in the particular layer. It is essential to control the density and ratio between the populations of cells that are applied to fabricate the skin construct. It is also crucial to determine the mechanical strength, porosity, and degradation rate of bioprinted construct. The desirable skin equivalent should be porous to provide the appropriate cells’ aeration. The pores should be interconnected to allow cells to attach. In addition, they should be of small size in order to protect from microbials.^102^ The desirable skin equivalent should have a pore size between 200 and 400 μm.^103^ Furthermore, they should be biodegradable and should maintain their 3D structure for minimum 3 weeks to enable the ingrowth of fibroblasts and blood vessels and to proliferate epithelial cells.^104^

## 6 Overview of 3D Skin Bioprinting Studies

In the past years, significant progress has been observed in the field of skin bioprinting.^51,52,97^ The studies on fabrication of skin equivalents started from printing only dermis,^81,92^ then the next two layers (epidermis and dermis) were generated,^52,86,95^ and subsequently trilayers (epidermis, dermis, and hypodermis)^93,96^ were obtained. Table 2 summarizes the most important studies on the fabrication of skin equivalents using bioprinting technology. Some details concerning the selected approaches are presented in this paragraph.

**Table 2:** Selected 3D Skin Bioprinting Studies

biomaterials/bioink	cell types	bioprinting method	main findings	ref
Collagen	NIH3T3 fibroblasts, human keratinocytes	Laser-based	Fabrication of viable skin constructs, formation of multilayered epidermis within 11 days.	(56)
Collagen type-I on Matriderm	Human immortalized keratinocyte, NIH 3T3 fibroblasts	Laser-based	Histological analysis: high density of fibroblasts and keratinocytes, expression of laminin protein (a component of basement membrane in the skin).	(59)
Collagen type I	Fibroblasts, keratinocytes	Extrusion based	Densely packed cells in epidermis layers and low density of cells in the dermis.	(45)
Collagen hydrogel precursor	Fibroblast, melanocytes, keratinocytes	Extrusion based	Fabrication of full-thickness skin model containing pigmentation.	(91)
Collagen and fibrinogen	Amniotic fluid-based stem cell or mesenchymal stem cells	Inkjet	The presence of blood vessels in the subcutaneous adipose tissue revealed in histological analysis.	(109)
Hydrogel fibrinogen and collagen type I	Fibroblast, keratinocyte	Inkjet (in situ)	Design of a system for in situ skin bioprinting. Acceleration of wound regeneration by bioprinted fibroblasts and keratinocytes compared to the controls.	(110)
Collagen hydrogel, gelatin, PCL (polycaprolatone)	Fibroblast, keratinocyte	Extrusion and inkjet based	Fabrication of skin model with functional transwell system containing stabilized fibroblast-stretched dermis and stratified epidermis layers	(108)
Gelatin, Fibrinogen, alginate	Fibroblasts, keratinocytes	Extrusion based	Generation of a full-thickness akin by scaffold-free bioprinting strategy.	(43)
Plasma-derived fibrin	Human fibroblast, human keratinocyte	Extrusion based	The structural and functional features and consistency of bioprinted skin are comparable to human skin.	(50)
Skin differentiation medium, Collagen I, fetal bovine serum,	Human keratinocytes, human fibroblast, human endothelial cells, human pericytes	Extrusion based	Fabrication of multilayered vascularized bioengineered skin graft biologically and morphologically similar to native skin.	(95)
Collagen, Thrombin, Fibrinogen	Neonatal human dermal fibroblasts and epidermal keratinocytes, dermal microvascular endothelial cells	Inkjet-based	Bioprinted scaffold revealed 17% better wound contraction	(111)
Gelatin, Glycerol, Fibrinogen, Hyaluronic acid, Poly(urethane)	Human fibroblasts, Human keratinocytes	Extrusion based	Development of 3D printed BioMask for facial skin regeneration. Histological analyses revealed the regeneration of skin tissue on complex wounds.	(112)
Fibrinogen, Glycerol, Gelatin, Hyaluronic acid, Aprotinin	Human keratinocytes, Human melanocytes, Primary human fibroblasts, follicle dermal papillary cells, preadipocytes	Extrusion based	The bioprinted skin enhanced the closure of the wound by promoting the formation of the epidermal barrier.	(93)

Pourchet et al.^43^ fabricated a two-layered skin substituent using a bioink mixture of gelatin and fibrinogen. The thickness of this construct was 5 mm. After 26 days of culture, the 3D printed skin revealed the histological features of native skin. In turn, Cubo et al.^50^ developed a full-thickness human skin using fibroblasts and keratinocytes embedded in human plasma with fibrinogen. Both in vitro and in vivo results revealed that the bioprinted skin equivalent resembled the native human skin and both dermis and epidermis layers were clearly identified. Lee et al.^45^ fabricated a two-layer skin equivalent by using keratinocytes and fibroblasts as constituent cells of the epidermis and dermis. The collagen was applied to form the skin dermal matrix. The histology and immunofluorescence studies showed that 3D printed skin constructs were morphologically and biologically similar to human native skin. However, some studies proved that biomaterials based on collagen have poor printability and long cross-linking time. Therefore, Ng et al.^105^ obtained polyelectrolyte-gelatin-chitosan hydrogels and reported that they had good biocompatibility with fibroblast skin cells and appropriate printability at room temperature. In turn, Rimann et al.^106^ reported an all-in-one solution for the fabrication of soft tissue skin models using bioprinting process with human primary fibroblasts and keratinocytes. In another study, Yanez et al.^107^ employed the 3D bioprinting technology to integrate capillary-like endothelial networks into a dermo-epidermal skin graft including neonatal human epidermal keratinocytes and neonatal human dermal fibroblasts. Moreover, histological characterization of obtained constructs demonstrated the formation of dermal and epidermal skin layers comparable to the native skin, which is accompanied by the presence of new microvessels in the mouse tissue. Min et al.^91^ elaborated the procedure of developing thick skin with pigmentations containing melanocytes. In turn, Kim et al.^108^ proposed a novel single-step 3D cell-printing using a functional transwell system. A hybrid approach was developed which involved extrusion and inkjet modules simultaneously. The construct based on collagen with polycaprolactone mesh (that inhibited the collagen contraction during maturation of tissue) was applied in this procedure. The skin model obtained exhibited promising biological properties. It contained steady fibroblast-stretched dermis and thick epidermis layers. Moreover, it was proved that due to this method, the costs and time consumption were lower compared to the stereotyped culture. Next, Hakimi et al.^40^ developed a hand-held skin printer allowing in situ formation of skin tissue sheets of different homogeneous and architected compositions. They also demonstrated that this system is compatible with dermal and epidermal cells incorporated with ionic cross-linkable alginate, enzymatically cross-linkable proteins, and their mixtures with collagen type I and hyaluronic acid. Admane et al.^52^ obtained a full-thickness human cell-based skin equivalent that exhibited structural, mechanical, and biomechanical properties similar to human skin. They fabricated the unique undulated pattern of the dermal-epidermal junction. Due to the great advances in 3D bioprinting presented above, the researchers started to search for the possibility of applications of skin equivalents that will be presented in the next paragraph.

## 7 Application of 3D Bioprinting in Skin-Related Research

Human bioengineered skin substitutes may be used for different clinical and research applications.^30,113−116^ With spreading interest in cosmetic/aesthetic procedures and rising rates of obesity, diabetes, and aging populations, the repair of damaged or lost tissue is a worldwide concern, and the demand for skin biofabrication is still growing. It is postulated that skin bioprints represent an alternative approach for the following:

- Regenerative medicine clinical applications (chronic wounds, burn injuries, ulcerations, reconstructive surgery after large oncological resections).
- Modeling physiological/pathological conditions (wound healing, UV response, aging, permeability of skin barrier, drug reaction, photoirradiation, skin cancer, genodermatoses, inflammatory conditions).
- Cosmetic/pharmaceutical industry (safety and efficacy of active agents, drug absorbance, drugs metabolization, personalized therapies).

**Figure 2:**
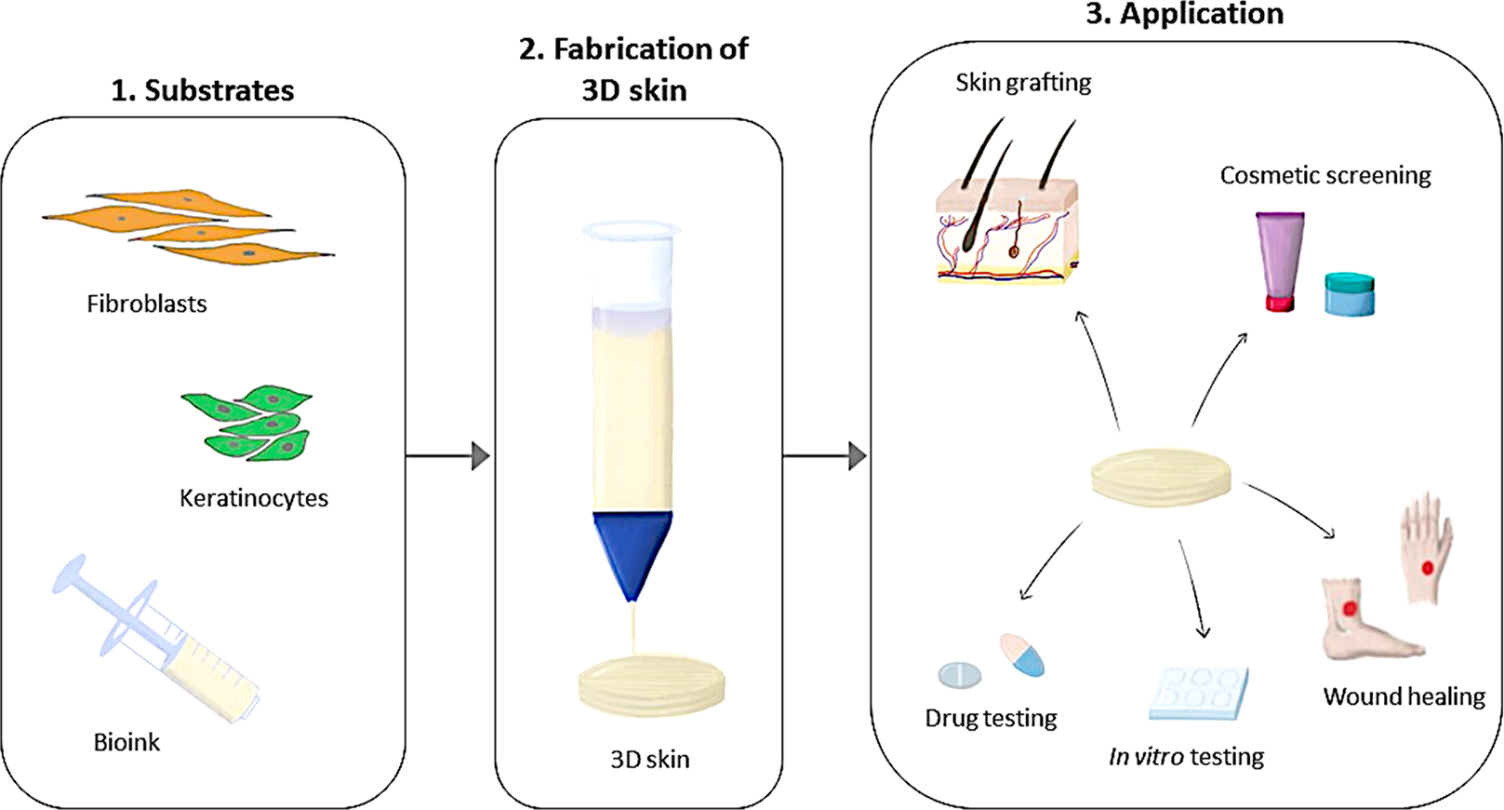
Overview of 3D skin bioprinting concept.

Also, the models of bioprinted skins may serve as a platform for the development of new formulations. Some legal conditions and regulations and ethical reasons related to the tests of safety and efficacy of new formulas in animal models by the cosmetic and pharmaceutical industry force the search for new solutions in the field of cosmetology, pharmacy, and medicine. Moreover, ex vivo skin represents a valuable model for skin penetration studies, but due to logistical and viability limitations, the development of alternatives is required. On the other hand, the traditional 2D cell culture has essential limitations, thus innovative technologies such as 3D bioprinting are needed. Figure 2 illustrates the 3D skin fabrication process and the main applications of this technology.

### 7.1 Treatment of Burn Injuries and Wound Healing

A lot of people suffer from nonhealing skin wounds. Traditionally, transplants from patients’ bodies or from donors are used to treat skin injuries. 3D bioprinting could be applied as an alternative for the above-mentioned method. The main advantage of this innovative technology is that the skin equivalents can be easily created in lesser time and cost.^4^ 3D bioprinting gives an opportunity to revolutionize the way of treatment in injury and surgery. Especially it can be useful to heal the burned skin. 3D bioprinters were created that provide an opportunity to print skin for injured patients.^4^ Two strategies such as ex vivo and in situ bioprinting are applied to fabricate skin for wound healing treatment. In ex vivo methods (inkjet-, extrusion-, laser-based bioprinting), a skin construct containing dermis and epidermis is printed, and next if necessary it is matured in vitro. Afterward, it is grafted to the wound of the patient. The simplest and the quickest ex vivo method is extrusion-based bioprinting. In this technique all components (such as human fibroblasts, human plasma, calcium chloride) necessary to form the dermis are deposited at the same time. Afterward, on the top of this layer, human keratinocytes are placed to create an epidermis. Michael et al.^56^ used laser-assisted bioprinting to develop skin equivalents and transplanted the mice’s wounds. After 11 days, the transplant adhered to the tissues located around the wound; in addition, the cells in the graft proliferated and differentiated. Cubo et al.^50^ demonstrated the suitability of a 3D bioprinter and primary human fibroblasts and keratinocytes to produce a human-plasma-derived bilayered skin to treat burn injuries and traumatic and surgical wounds. Xiong et al.^117^ reported that the rate of wound healing increased by using 3D printed gelatin-silk fibroin composite scaffolds. The addition of fibroblast growth factor might improve the treatment effectiveness. In turn, Lian et al.^118^ added to hydrogel (that contained gelatin, sodium alginate, gelatin methacrylate) normal human dermal fibroblasts and normal human keratinocytes to fabricate a skin substituent that was applied to reduce scars in nude mice. The bioprinted skin revealed much better results in healing the wound than the bioprinted hydrogel or untreated wound control. The histology and immunofluorescence analyses performed 28 days after grafting showed that the thickness of both dermis and epidermis was comparable to that of mice. Additionally, the microvascular formation in the dermis layer was also detected.

In turn, in an in situ bioprinting approach, the skin cells suspended in hydrogels are directly printed on the injured part of the patient’s body. Subsequently, the cross-linking of the bioinks is performed to reproduce the 3D skin structure.^33^ Binder et al.^26,110^ created a computer software and bioprinting tool that consisted of a cartridge delivery system composed of a series of inkjet nozzles and laser scanner. On the basis of the data acquired from the laser, the 3D model of the wound was reconstructed. In the next step, the printing heads filled dropwise the wound with bioink composed of fibroblasts, collagen I, and fibrinogen. At the same time, thrombin was added which is required to cross-link fibrinogen into a fibrin hydrogel. In the last stage, keratinocytes were printed. The experiments performed on the nude mice proved that the wound was repaired by printed skin within 3 weeks, which was faster than the controls (5 weeks). This method is original and promising, but it is still at the developing stage and more trials are required.

Skardal et al.^119^ created a special type of bioink (photocrosslinkable heparin-conjugated hyaluronic acid) that was capable of releasing cell-secreted growth factors. This complex system was dedicated for in situ skin printing and tested in wound healing treatment. The bioink and amniotic fluid-derived stem cells were printed directly on the wound of the murine model. Afterward, with the usage of thiol–ene photopolymerization process under exposure of ultraviolet light, the bioink was cross-linked. Wounds treated with the presented above procedure revealed a higher closure rate compared to nontreated control. In turn, Albanna et al.^41^ reported a new type of mobile skin bioprinting procedure that quickly healed the complex injuries. The biomaterials included fibrinogen and thrombin. The immunohistochemistry analysis of human cells showed that human fibroblasts, keratinocytes, and endogenous cells were present in the skin layers. The authors also proved that the treatment of wounds with autologous fibroblasts and keratinocytes, which were applied immediately to the target place, improved the wound healing process. The performed studies proved that the cells (such as keratinocytes, fibroblasts, melanocytes) isolated from patients can be applied during the bioprinting process. After in vitro culturing, the cells can be mixed with appropriate biopolymer and printed to obtain a skin construct that after maturation can be implanted into the injured area of the patient.

The main limitation of 3D bioprinting technology regarding wound healing treatments is that the time required to obtain sufficient autologous cells to fabricate a large skin surface is not diminished sufficiently yet. It is essential to mention that the patients who suffer from extensive burns require treatment in as short of a time as possible. Therefore, the immediate application of bioprinted skin equivalents is essential to accelerate the wound recovery and decrease the hypertrophic scar tissue.^120^

### 7.2 Modeling of Skin Diseases

3D tumor models may help to analyze the mode of action in cancer proliferation and metastasis and reaction to the selected drug. The bioprinted tissues can be combined with tumor cells to obtain the new model of diseases. Thus, melanoma was introduced to the human in vitro skin equivalent.^121^ Liu et al.^97^ fabricated skin tissues to generate disease models of Atopic Dermatitis (AD). Several characteristic features of AD were distinguished in these models such as hyperplasia and spongiosis; elevated level of proinflammatory cytokines; early and terminal expression of differentiation proteins. This study revealed that bioprinting can be applied to fabricate human skin substituents with different types of cellular complexity for modeling a certain disease. This method gives an opportunity to understand the mechanisms of various pathologies.

### 7.3 The Cosmetic and Pharmaceutical Industry

In light of the entry into force of the EU Cosmetic Regulation (EU/1223/2009) with the complete ban of animal testing for cosmetic purposes, there is a strong demand to obtain skin equivalents that could serve as an alternative to animal trials. It should be added that the use of animal models is not only restricted due to ethical reasons but also due to their incomplete similarity to human skin. Therefore, the research results in some cases are not clear enough.^122^ The human physiological system is different than the animal one. Consequently, ca. 50% of drugs that passed positively the animal trials proved to be toxic for humans and inversely.^123^ The worldwide trend in both pharmaceutical and cosmetics industries is to search for skin models that could be applied to test new substances and novel topical formulations.^124,125^

Therefore, 3D bioprinting has attracted the blooming attention of skincare companies. It is expected that this new technology may revolutionize the testing of cosmetic and topical products. As it was presented above, skin is multilayered and contains various cell types. 3D bioprinting gives the opportunity to deposit cells in this arrangement. 3D bioprinted skin may bring a lot of advantages for both cosmetic and pharmaceutical industries. Before clinical studies of each new substance/drug, their safety should be examined in in vitro tests. The pharmaceutical/chemical companies may test the medicines and chemicals by applying skin models fabricated using 3D bioprinters,^29^ whereas cosmetic formulations must be assessed for potential toxic and allergic effects prior launching to the market.^30^ Therefore, 3D bioprinted skin may be considered as an appropriate platform to perform assessment and screening of cosmetic and pharmaceutical formulations. Due to this technology the drug and product testing could be faster, cheaper, and more effective. In addition, it can be more ethical. The method can be fully standardized and automated, thus the production costs will be reduced. For cosmetic testing different types of skin such as normal, dry, oily, and sensitive should be fabricated.^126^ In addition, the 3D skin bioprinting has the potential to be applied to study drug/active compound penetration and absorption through the skin. This technology attracted the attention of global cosmetic leaders such as L’Oreal and Proctor & Gamble, who invested in the research and development of 3D bioprinted skin models.^127^

### 7.4 Clinical Application of 3D Skin Bioprinting

The translation of skin bioprinting from academic research to clinical practice is promising. Different forms of potential clinical applications involving regenerative medicine like cell therapy (cell-based immunotherapy, stem cell therapeutics) and tissue engineering were found^4,41,128−130^ 3D bioprinting may be used for the regeneration of skin tissue and appendages. In light of this, one of the most important clinical needs is skin grafts. The print of skin biological scaffold may serve as an alternative to painful traditional skin grafts to minimize donor requirements and provide better treatment of skin grafting.^4,41^ Moreover, this technology can be used to treat chronic and nonhealing wounds such as diabetic, venous, or pressure ulcers and burn wounds.^41^ Günther et al.^40^ developed hand-held 3D bioprinting instruments that ameliorated healing in porcine models of full-thickness burns. The system promotes the skin regeneration and reduces scars; therefore, it has potential to be introduced in clinical settings in the near future. In addition, the skin bioprinting may also revolutionize aesthetic medical procedures. 3D skin bioprinting has the potential for reconstituting the cancer microenvironment.^4,130^ It can be used to create tumor models from patients’ cancerous cells, which can be further helpful for the personalization of anticancer drugs. Furthermore, this procedure may serve as a powerful tool for studying various biochemical pathways’ roles in carcinoma initiation and progression.^130^ Another clinical application of 3D skin bioprinting is precision medicine.^4^ In light of this, it can be used for providing individualized medication as per the genetic profile and health condition of the patient. In addition, personalized skin bioprinting is pointed out as one of the promising techniques of tissue engineering for astronauts in future, long-distance space missions.^131^ However, despite these great perspectives, we should be aware that skin bioprinting is still in its clinical infancy. The automated procedures need to be adopted in order to efficiently translate bioprinted skin to the clinical settings. Multiple experimental, ethical, budgetary, and regulatory difficulties hinder its rapid clinical application.^132^

## 8 Advantages and Limitations of 3D Bioprinting

Due to the bioprinting technique, it is possible to produce 3D skin models in an automated way, which is faster than manual methods. During the skin fabrication process, there is an opportunity to introduce different molecules and cells that promote pigmentation, vascularization, and innervation, which enable us to create biomimetic equivalents.^133^ 3D bioprinting allows the precise deposition of different cells and biomaterials with high reproducibility and flexibility.^22^ The skin constructs developed using this method have good plasticity, extensibility, and can be printed in high yield.^120^ Therefore, the main advantage of skin bioprinting is the development of clinically relevant skin constructs that closely mimic the native skin architecture and heterogeneity via precise positioning of multiple cell types. Large-scale fabrication is another benefit of 3D-bioprinting that could be favorable for the cosmetics and pharmaceuticals screening process. Furthermore, specific skin equivalents dedicated to the selected patients can be developed by printing autologous cells.^134^ This may contribute to developing personalized therapies for skin diseases.

Despite many advantages of 3D bioprinting, it is important to mention the obstacles that may be encountered during skin fabrication. The whole system is of high complexity. Therefore, specialized staff are required to carry out the production process. In addition, the 3D bioprinter is of a professional level and its maintenance is high cost. Therefore, the rapid promotion of the application of bioprinting technology could be limited. The challenges for skin bioprinting are primarily associated with selecting appropriate printable bioinks to support the function of cells and stimulate the fabrication of new ECM after printing. A critical issue is also to develop the large skin equivalent with highly developed vasculature. Some researchers have worked on fabricating the multiscale vascular networks including dendritic channels^135^ and straight pipeline;^136^ however, they were still far from the blood vessels of native skin. Another bottleneck of bioprinting concerns the difficulty to fabricate the skin constructs that contain hair follicles, sweat glands, and sebaceous glands. An important challenge is also to fabricate the skin with the appropriate color and texture that mimic the native skin. Furthermore, cell viability may be affected by different factors such as bioprinting method applied, the printing speed, and types of seeding cells.^37,105,106^ Furthermore, the heat that is generated while printing may damage the cells. Another limitation is related to patient safety. The skin 3D bioprinting process is not yet mature. Therefore, some security concerns may occur in the future concerning safety problems when the bioprinted skin will be directly applied to patients in clinical studies. There are also legal challenges that need to be taken into consideration before the product can be released to the market.^137−139^

## 9 Conclusions

3D bioprinting can bring different advantages in various fields. It can eliminate the need for donors of organs. Moreover, this technology may improve the drug discovery process. Additionally, it may eliminate animal testing. The main challenge seems to be the creation of functional skin with sufficient vascularity, innervation, and functions such as touch sensation and perception.^29^ In addition, the color, texture, and individual traits of native skin are other difficulties. An upcoming direction is to generate more complex skin models. Future perspectives also involved producing dry, oily skin with different textures, pigmented with various shades/tones. It should be noted that there are some ethical, social, and legal challenges requiring attention before the technology and product may be successfully used in a large scale and enter the clinical world.
